# Social Support for Self-Care: Patient Strategies for Managing Diabetes and Hypertension in Rural Uganda

**DOI:** 10.5334/aogh.3308

**Published:** 2021-08-19

**Authors:** Andrew K. Tusubira, Christine K. Nalwadda, Ann R. Akiteng, Evelyn Hsieh, Christine Ngaruiya, Tracy L. Rabin, Anne Katahoire, Nicola L. Hawley, Robert Kalyesubula, Isaac Ssinabulya, Jeremy I. Schwartz, Mari Armstrong-Hough

**Affiliations:** 1 Uganda Initiative for Integrated Management of Non-Communicable Diseases, Kampala, Uganda; 2 Department of Community Health and Behavioural Sciences, Makerere University, College of Health Sciences, Kampala, Uganda; 3 Department of Internal Medicine, Yale School of Medicine, New Haven, CT, USA; 4 Yale Network for Global Non-Communicable Diseases, Yale University, New Haven, CT, USA; 5 Department of Emergency Medicine, Yale School of Medicine, New Haven, CT, USA; 6 Child Health and Development Centre, Makerere University, Kampala, Uganda; 7 Department of Chronic Disease Epidemiology, Yale School of Public Health, New Haven, CT, USA; 8 Departments of Physiology and Internal Medicine, Makerere College of Health Sciences, Kampala, Uganda; 9 African Community Center for Social Sustainability (ACCESS), Nakaseke, Uganda; 10 Uganda Heart Institute, Mulago Hospital Complex, Kampala, Uganda; 11 Department of Internal Medicine, Makerere College of Health Sciences, Kampala, Uganda; 12 Department of Social & Behavioral Sciences, School of Global Public Health, New York University, New York, NY, USA; 13 Department of Epidemiology, School of Global Public Health, New York University, New York, NY USA

## Abstract

**Background:**

Low-income countries suffer a growing burden of non-communicable diseases (NCDs). Self-care practices are crucial for successfully managing NCDs to prevent complications. However, little is known about how patients practice self-care in resource-limited settings.

**Objective:**

We sought to understand self-care efforts and their facilitators among patients with diabetes and hypertension in rural Uganda.

**Methods:**

Between April and June 2019, we conducted a cross-sectional qualitative study among adult patients from outpatient NCD clinics at three health facilities in Uganda. We conducted in-depth interviews exploring self-care practices for hypertension and/or diabetes and used content analysis to identify emergent themes.

**Results:**

Nineteen patients participated. Patients said they preferred conventional medicines as their first resort, but often used traditional medicines to mitigate the impact of inconsistent access to prescribed medicines or as a supplement to those medicines. Patients adopted a wide range of vernacular practices to supplement treatment or replace unavailable diagnostic tests, such as tasting urine to gauge blood-sugar level. Finally, patients sought and received both instrumental and emotional support for self-care activities from networks of family and peers. Patients saw their children as their most reliable source of support facilitating self-care, especially as a source of money for medicines, transport and home necessities.

**Conclusion:**

Patients valued conventional medicines but engaged in varied self-care practices. They depended upon networks of social support from family and peers to facilitate self-care. Interventions to improve self-care may be more effective if they improve access to prescribed medicines and engage or enhance patients’ social support networks.

## Background

Non-communicable diseases (NCDs) account for the majority of the global burden of disease, and that burden is higher in low- and middle-income countries (LMIC) [1,2]. NCDs such as diabetes and hypertension threaten resource-limited countries even as they continue to manage a substantial burden of infectious diseases [1,2,3]. Diabetes and hypertension have similar risk factors and commonly co-occur; both conditions are associated with high and lifelong costs of care [4,5]. The economic burden of these NCDs is particularly high for individuals and families in rural settings [6,7,8]. LMICs must identify cost-effective approaches to manage their growing burden of diabetes and hypertension, with special attention to rural populations. One such approach is to strengthen self-care practices among those living with NCDs.

Self-care is a patient-driven process involving activities intended to manage symptoms and maintain physiological stability [9]. Self-care includes all actions patients take to attain and maintain good health [10]. Conventionally recommended self-care practices for patients with hypertension or diabetes include a diet rich in fruits and vegetables, adherence to medication, regular physical activity, smoking cessation, weight loss, and moderation of alcohol consumption [11]. These practices can significantly improve health outcomes for patients with NCDs, reducing the cost of care [12].

Social support is an important driver of self-care for diabetes and hypertension [13]. Social support may take the form of physical, financial, or psychological help from family, friends, and community members [14]. It may consist either of emotional support (i.e., a confidant) or instrumental support (i.e., tangible and/or physical assistance). For patients with chronic diseases, such support has been demonstrated to build resilience and increase confidence to perform or sustain self-care practices [15].

However, little is known about the self-care practices of patients living with diabetes and/or hypertension in LMIC. Moreover, the relationship of care practices to rural patients’ social and cultural contexts is under-addressed in the literature. We therefore examined how patients engaged in self-care practices for diabetes and hypertension in rural Uganda, where the prevalence of these conditions is increasing, most people living with diabetes experience suboptimal glycemic control, and most people living with hypertension experience suboptimal blood pressure [16,17,18].

## Methods

### Study design

We carried out in-depth interviews with patients to explore practices and resources for attaining and maintaining health in light of diagnosis with diabetes and/or hypertension.

### Study setting

People living with NCDs in Uganda seek varied channels of care and exhibit poor adherence to conventional treatment regimens [7]. This study was set at three health facilities in Nakaseke, a rural district in central Uganda. Nakaseke has Uganda’s highest hypertension prevalence at 28.5% among adults [16]. Nakaseke General Hospital, Semuto Health Center-IV, and Nakaseke LifeCare Medical Center host specialized clinics for diabetes and hypertension: Nakaseke General Hospital serves approximately 75 patients, Semuto 10 patients, and LifeCare 15 patients at these weekly clinics. There, a nurse or clinician checks weight, blood pressure, and blood sugar; delivers health education; prescribes medications; and schedules monthly follow-up appointments. Medications are dispensed at a clinic-based pharmacy.

### Study population

We included adult patients who 1) attended outpatient NCD clinics at one of the three selected facilities and 2) were diagnosed with diabetes and/or hypertension at least three months prior.

### Sampling

We purposively selected participants for maximum variation by condition and gender, selecting at least two patients with hypertension, two with diabetes and two with both conditions from each facility. We selected at least one male and one female for each condition category. We initially selected 18 participants. During interviews, we learned about the phenomenon of informal patient group leaders. We therefore selected the nineteenth participant to include at least one patient leader. Research assistants approached patients in the waiting area to inform them about the study prior to receiving care. After the patient completed their visit, they were invited to interview.

### Data collection

Three (one male, two female) trained research assistants with backgrounds in social science interviewed participants using a semi-structured interview guide between April and June 2019. The guide was developed and reviewed by the research team and pretested among four patients with diabetes and hypertension. After pretesting, we revised study instruments to improve clarity and alignment with the overarching research question.

The interview guide (Appendix 1) included five key domains: perspectives on their condition(s), experiences receiving care, challenges to medication adherence, symptom recognition and responses, and practices intended to prevent adverse conditions or be well. All interviews were conducted in-person in the local language, Luganda, and lasted 30–45 minutes. Participants were compensated 15,000 Ugandan shillings (UGX), equivalent to $4 United States Dollar (USD) at the time of the study, for their participation. Audio recordings were transcribed verbatim in Luganda and translated to English.

### Analysis

We used a conventional approach to thematic content analysis to inductively derive categories [19]. A team of four researchers, including AKT and MAH, independently read and coded three transcripts to develop initial codes. Coders discussed and reached consensus about the initial code structure, then applied it to further transcripts while remaining open to new codes. Another meeting was held to finalize the structure. The resulting codebook was applied to all transcripts using Atlas.ti 8.5. Emerging findings were refined through works-in-progress sessions as part of a training grant (NIHD43TW009607-07) and review with co-investigators.

### Human subjects

We received approval from the Makerere University School of Medicine Ethics Review Committee, Yale Human Subjects Committee, and Uganda National Council of Science and Technology (HS 2698). Health facilities provided administrative clearance. All participants provided written informed consent.

## Results

We enrolled all 19 patients invited to participate (***Table 1***). Their mean age was 55 years (standard deviation ±12). Most were farmers (n = 10, 53%) and had attained at least primary-level formal education (n = 17, 89%). Six (32%) participants were living with hypertension, six (32%) with diabetes, and seven (36%) with both conditions.

**Table 1:** Participant characteristics.


CHARACTERISTIC	FREQUENCY	PERCENT

*Age group*		

35–49	5	26%

50–59	8	42%

60 and above	6	32%

*Gender*		

Male	10	53%

Female	9	47%

*Education*		

No education	2	11%

Primary	8	42%

Secondary	7	37%

Tertiary	2	11%

*Occupation*		

Peasant farmer	10	53%

Business	5	26%

Other	4	21%


Three overarching themes emerged. First, patients preferred prescribed medicines as their first resort. However, they used traditional medicines to mitigate the impact of inconsistent access to prescribed medicines or as a supplement to those medicines. Second, patients adopted a wide range of vernacular practices to supplement treatment or self-monitor in the absence of basic equipment or materials. Finally, patients relied on social support from family members and patient peers to mitigate the impact of uncertain access to prescribed medicines.

### Patients preferred conventional medicines but used traditional medicines

Patients reported that clinician-prescribed conventional medicines were their key resource for self-care. However, their ability to adhere to prescribed medicines was limited by inconsistent access. One explained:

*In order to survive, I have to do what [clinicians] told me and remain on medication. But the main challenge we have is getting medicines. So I sometimes miss taking the medicines*. (IDI-8, HTN-DM, Female, 40)

Because they lacked consistent access to inexpensive medicines from public or nonprofit health facility pharmacies, patients said they had to “improvise” by visiting private facilities:

*I adhere to them and I take my medication as prescribed by the doctor, only that the medicines we receive are not enough for a month. We just improvise and buy in order to maintain our health*. [Clinicians] *normally tell us to go and buy the medicines, yet the medicines are very expensive*. (IDI-4, HTN-DM, Male, 65)

When patients bought medicines at private pharmacies, they typically secured fewer doses than prescribed.

Unable to access medicines at public facilities or afford those at private pharmacies, many patients sought other remedies. Some described using traditional remedies, including local herbs, bitter vegetables, and saltwater, in place of unavailable or costly prescribed medicines. Patients perceived that these remedies would prevent complications, reduce their blood pressure, stabilize blood sugar, and/or relieve pain in the absence of conventional medicines (***Table 2A***). Many saw herbal remedies as a stopgap for times when they were unable to obtain prescribed medicines:

**Table 2A:** Practices in place of missed medicines (when conventional medicines are not available).


ACTION/PRACTICE	REASON OR TRIGGER (SYMPTOM OR SIGN)	INTERPRETATION OF TRIGGER	PERCEIVED OUTCOME

Soak feet in salt water	Swelling of feet	High BP	Prevents likely complication

Sleep on a cold surface	Burning sensations in body	High BP	Relieved

Drink cold water	Heat in the body and feet	High BP	Cooled body

Undress or sleep on a cold surface	Heat in the body and feet	High BP	Cooled body

Eat raw carrots	Itchy feeling/tingling	High BP	Partial relief

Use herbal teas or herbal coffee	Chest pain, joint pains	High BP	Partial relief

Drink water	Palpitations	High BP	Psychological relief

Take herbal medicines	No medicines at expected time for swallowing the medicines	High BP	Reduced BP

Take oral rehydration salt (ORS)	No medicines at expected time for swallowing the medicines	High sugar	Temporary relief

Walk around	Numbness of feet or toes	High sugar	Reduced numbness

Take herbal medicines	Erectile dysfunction	High sugar	Substitute medicines

Eat bitter tomatoes	Itching	High sugar	Partial relief

Take oral rehydration salt (ORS)	No medicines at expected time for swallowing the medicines	High sugar	Partial relief

***I: Are there things you do when you have failed to get the recommended dose?***

*R: Yes, I use herbals such as stem pieces of bitter trees. I boil them and drink. I also get kasaana* (Acacia hockii) *tree roots, which I boil and drink… There is a time I failed to raise money and resorted to using these herbs in order to prevent getting an attack due to this disease*. (IDI-10, HTN-DM, Female, 59)

Similarly, patients described using traditional medicines to ration insufficient doses of prescribed medicines:

*R: Sometimes when I fail to get all the medicines, I sparingly take them along with herbs*.

***I: Can you please clarify?***

*R: If* [pharmacists] *have given me tablets, say, for two weeks and they told me to take two tablets thrice a day, what I normally do is to swallow two tablets twice a day, omiting these two tablets and push[ing] them forward. So instead of swallowing these two tablets during the day, I drink some herbal medicine during day. If I realize that my medicine is reducing and yet there are more days, I reduce and swallow one tablet in the morning and one tablet in the evening. So at around lunchtime, I drink my cup of herbal medicine which I have prepared*. (IDI-15, DM, Female, 36)

When patients were able to obtain all prescribed doses, some supplemented prescriptions with traditional medicines. They used traditional medicines to attain a desired health status, such as weight loss, relief from pain, or because they hoped to cure their condition (***Table 2B***). One patient described finding relief through a costly herbal concoction:

**Table 2b:** Use of traditional medicines alongside conventional medicines (when conventional medicines are available).


ACTION/PRACTICE	REASON OR TRIGGER OF (SYMPTOM OR SIGN)	INTERPRETATION OF TRIGGER	REPORTED OR PERCEIVED OUTCOME

Takes traditional medicines	Palpitations, Swollen face, heavy eyes	High BP	Partial relief

Takes traditional medicines	Feeling weak	High sugar	Partial relief

Adds traditional herbal condiment to food	Weight gain	Lots of fat in the body, clogged blood vessels	Breaks down fat, herbs cleanse the veins

Takes locally prepared herbal mixtures	Told that traditional herbs cure	Desire for cure	Herbs may cure

Decocts and takes herbal teas or coffee or syrups	Severe headache, joints pains, chest pain	High BP	No effect but some relief

Takes Arabic herbal medicines	Feeling dizzy	Low sugar	Raised sugar level

*R: I have used some herbal medicine from a certain Hajjat. She insists that she heals diabetes and she really has medicine that heals diabetes. I even bought a jerry can worth UGX80,000*[$22 USD], *but doctors say that diabetes is incurable. … It really worked for me and gave me some relief. For instance, my sugar level was at 17*[mmol/l, equivalent to 306 mg/dl*] and after drinking it reduced to 15*[270 mg/dl]*, 12*[216 mg/dl], *11*[198 mg/dl] *like that. I have not yet got enough money to buy more, she just gave me a mixed jerry can*.

***I: Did you use it because she said it heals diabetes?***

*R: I used it because I heard her on radio saying she really heals diabetes. It is so helpful, but she is expensive. I plan on buying it again when I get money, because it was of great help to me: it brought relief to me*. (IDI-11, DM, Male, 52)

### Patients adopted many vernacular self-care practices

Besides using medicines, patients restricted diet, sought ways to control stress, and devised self-monitoring strategies in order to maintain their health. They adopted a variety of self-care practices in response to specific symptoms, such as walking barefooted for relief when their feet were numb, soaking swollen feet in salt water, and massaging with warm water to relieve itching (***Table 3***). Many restricted their diet:

*As a result of this disease, I decided to desist from soft drinks and all sweet things. I no longer take sugar, but life is not enjoyable. I eat mostly matooke [steam-cooked starchy bananas] and Irish potatoes. For cassava, I eat just a piece when I feel like. I mainly avoid fatty foods such as margarine and fatty meat. I, however, eat roasted meat*. (IDI-5, DM, Male, 65)

**Table 3:** Practices to supplement treatments.


ACTION/PRACTICE	REASON OR TRIGGER (SYMPTOM OR SIGN)	INTERPRETATION OF TRIGGER	REPORTED OR PERCEIVED OUTCOME

Takes time off from people	Stressed or unhappy	Causes BP or sugar levels to increase	Feels better

Swallows some anti-biotic	Feeling dizzy	High BP	Gets some relief

Avoids worrying, takes off time to sleep	Lack of strength, general body weakness, palpitations	High BP	Feels better, palpitations stopped

Avoid eating food (fasting)	Hearing the heart beats	High BP	To reduce weight

Takes a walk	Feeling weak	High BP	To improve blood circulation

Swallows some painkillers (analgesic)	Swelling of feet, numbness of feet or legs	High BP	Swelling reduced, feels better

Takes a walk	Breathing heavily	High BP	To sweat and reduce fat

Drinks lots of water	Constant headache, constantly feeling thirsty, frequent urination	High sugar	Gets some relief

Eats food and takes a walk	Cloudy vision	High sugar	Felt better

Increases the medicine dosage	When the urine is yellow	High sugar	Gets some relief

Massages legs with warm water, stretch legs morning and evening	Paralysis of feet and knees	High sugar	Perceived to help straighten blood vessels, gets some relief

Uses worm water	Itches	High sugar	Gets some relief

Walks barefooted on small stones	Numbness of feet	High sugar	Feels good

Digging, walking and household chores	Main physical activities	Improve blood circulation	Body functions well

Eats sugar or takes something sweet (e.g., sweet bananas)	Foaming in mouth, Cloudy vision, body weakness, or temporarily unable to speak	Low sugar	Stabilized, got better

Taste own urine	Anxious about sugar level	Sweet taste implies high sugar	Perceives the sugar level


When patients with diabetes felt weak or experienced symptoms they linked to low sugar level, many said they consumed sweet things to restore blood sugar:

*When I get a foam in the mouth to an extent that my tongue cannot move, I know that my sugar level is low. So, I lick some sugar or dissolve it in water and I drink. It is my first medicine. Even other diabetic patients have told me that I should take sugar the moment I notice that my sugar level has reduced*. (IDI-12, DM, Female, 78)

Because patients with diabetes were anxious to ascertain their sugar level but could not obtain glucometers, they improvised self-monitoring practices. For example, some reported tasting urine to gauge sugar level:

*R: Some of our colleagues [fellow support group patients] tell us that they check their sugar levels using their urine and that’s what I currently do. If the urine is very sweet, then the sugar level is high. But if it tastes like the salt of Lake Katwe, then the level is low*. (IDI-15, DM, Female, 36)

Patients saw these practices as adjuncts to conventional treatments and diagnostic tests to which they lacked access, rather than as preferred practices for maintaining health.

### Patients sought social support for self-care

Patients attempted to mitigate uncertain access to conventional medicines by drawing on other resources in their social environment. They typically saw their children as their most important source of emotional and instrumental support to sustain self-care. In particular, children were a primary source of instrumental support, providing money for medicines, transport, and necessities at home:

*The children buy the medicines and bring them. They can bring for me some medicines from Kampala where they stay. Sometimes children send money and I buy medicines*. (IDI-2, HTN, Female, 52)

Patients also looked to spouses for social support. Some husbands looked to their wives for emotional support and preparation of meals. Wives tended to look to husbands solely for financial support. These patients counted on their children for financial support for treatment when support from the spouse dwindled:

*R*: [The children] *help me. Because if I don’t have money for transport to come, they can mobilize for me*.

***I: Who are those?***

*R: The children we produce, because I may fail and my husband also fails. So we resort to asking our children for money*. (IDI-10, HTN-DM, Female, 59)

Patients considered their children a final but frequently-called-upon resort for financial resources to sustain supplies of medicine.

Finally, patients reported receiving peer support through unofficial patient groups. Some –mostly diabetes patients—described organizing themselves into groups for the purpose of mutual support. In addition to emotional support, patients received instrumental support in the form of cash assistance, transport, or medicines from other group members. One patient described how his patient group organized and pooled resources for mutual support:

*That group has helped more on the side of unity. Because we have the 1000UGX we collect every meeting but that money is for disaster preparedness, such that in case one of us is not able to come due to transport challenges, he calls the leaders and they send him transport instead of staying home and dying… The group still helps me especially on the medicines and [glucose monitor] strips. We collect 3000UGX[<$1 USD] per head so that in case government does not bring them, the doctors buy for us with our own money*. (IDI-5, HTN-DM, Male, 65)

In addition to material support, patients reported receiving informational support about self-care practices from other patients in their group:

*Our leaders in the group like that man* [patient member of support group]*, sensitize us on how we can protect ourselves, feeding patterns, and the way we are supposed to behave. For instance, I do not have to be with a gloomy face, I have to be happy*. (IDI-10, HTN-DM, Female, 59)

Groups were also a source of emotional support. Patients reported watching out for one another and creating expectations for self-care, food relief, and regular appointment attendance. A group leader explained:

*We don’t allow anybody in our group to miss an appointment without calling us to let us know why. You must show up and in case you won’t come due to transport issues, you call us and we send you money so you report. Now, in case someone is ill and they don’t have money, should we let them die? So we decided to collect 1,000UGX[<$1 USD] every time we come here. We also lend to that person who doesn’t have what to eat… And when you have lost a loved one, we give you condolence of 30,000UGX[$8.1 USD]*. (IDI-3, HTN & DM, Male, 76)

## Discussion

In this interview-based study of patients’ self-care for diabetes and hypertension in rural Uganda, we found that patients used both conventional and traditional medicines, but preferred conventional medicines as their first resort. They also adopted vernacular practices to supplement conventionally recommended self-care practices. Partly in response to inconsistent access to conventional medicines, patients frequently sought support from family and peers.

Crucially, we found that patients relied on networks of social support to mitigate uncertain access to medicines and meet costs associated with care. Studies of chronic HIV care in Uganda demonstrate that families, friends and other significant persons can be important sources of similar support for adherence to anti-retroviral therapy [20,21]. Similarly, a study in Nigeria identified families as the most accessible source of financial support for patients with hypertension and diabetes and found that this marginally impacted health outcomes [22]. Limited economic resources may increase the importance of such social networks for accessing treatment [8]. We add to this literature the notable finding that patients relied most on their children for support, rather than peers or spouses—even when they characterized their children as financially unstable or living outside the household. We identify a critical need for interventions to facilitate additional sources of instrumental and emotional support for patients.

We also found that many patients with diabetes participated in loosely organized patient groups. Patients pooled their own limited resources to provide test strips, transport support, emergency funds for medicines, and even food relief. While groups spontaneously arose in clinic waiting rooms, they were not affiliated with the health facilities. This is consistent with an earlier study in Uganda, which found that informal diabetes support groups helped patients manage frequent stock-outs of medicines [23]. Our study affirms the centrality of group support as a psychosocial and financial resource for managing health among rural patients. This finding is particularly important because it suggests a viable local model for a cost-effective, patient-centered intervention to mitigate resource variability. Patient-driven groups can provide additional sources of peer support for all patients. Such groups may be cost-effective for supporting and extending existing conventional chronic care delivery models, particularly among low-income and vulnerable populations [24]. For instance, Uganda’s differentiated service delivery platform, which offers facility-based groups for patients living with HIV, reduced total costs for antiretroviral drugs, laboratory commodities and health workforce needs [25].

Strengthening and scaling these groups for patients living with non-communicable diseases could address gaps in social support, decrease pressure on families, increase access to essential medicines and supplies, facilitate effective self-care, and improve adherence. Further research should develop and evaluate interventions to expand and strengthen the patient peer support model for rural settings, including mHealth tools to augment peer support. Building on the Centers for Disease Control’s Chronic Disease Self-Management Program and existing differentiated care models established for HIV in LMIC, patient group interventions could include components such as interactive education, symptom management activities, and mutual support for the costs of care [25,26].

We also found that patients valued prescribed medicines and overwhelmingly preferred them for disease management. Indeed, patients may even overestimate the capacity of these medicines. However, patients also reported that their access to prescribed medicines was poor or inconsistent. Insufficient medicine is the commonest reason for poor adherence to medication in Uganda [7,27], where public facilities are the primary source of free medicines for patients with NCDs. Recent work has demonstrated that the majority of doses prescribed for diabetes and cardiovascular disease at these facilities are not dispensed due to real or anticipated shortage [28]. Therefore, patients must obtain their prescriptions elsewhere, if at all [27,29]. New strategies are urgently needed to improve access to medicines; this, in turn, could improve adherence and reduce reliance on non-recommended care practices.

Because they had no test supplies, patients in our study devised their own self-monitoring strategies. Chronic care in Uganda is persistently affected by poor availability of essential materials and equipment [30,31,32]. Similar gaps in NCD service provision have been reported in other developing countries, like Malawi [33], Ethiopia [34], and Bangladesh [35]. There is urgent need for strategies to improve access not only to medicines but also to essential materials and equipment. Strategies to improve self-care in these settings must attend to the limited resources available for self-monitoring at home. Patient groups may mitigate the impact of poor access to essential materials by enabling patients to pool resources, including test supplies [23]. Additionally, patient group activities could potentially influence patient behavior to reduce dependency on traditional medicines.

Finally, we found that patients used traditional medicines in part to mitigate the negative impact of inconsistent access to prescribed medicines. The use of traditional medicines to compensate for rationed prescription medication may imply that patients perceive traditional treatments to play a similar role as prescribed medicines, as has been reported in other studies in Uganda and India [23,29,36]. Traditional medicines are common in Uganda and promoted by healers, peers, family members and media [7,23,29]. Notably, patients in our study did not report ceasing conventional medicine in favor of traditional medicine, as others have found in south-western Uganda [29]. Rather, patients in our study believed their prescribed medicines to be central to managing their condition. Additionally, patients in this study relied on biomedical monitoring to judge the effectiveness of the traditional medicines they consumed.

This work should be regarded within the context of some limitations. Most notably, we recruited participants from health facilities. Participants may face fewer barriers to accessing care or have different health-seeking behaviors than the general population. Findings may not reflect experiences or practices of people not engaged in clinical care. Future research should examine patterns detected through this exploratory work in larger, representative samples. For instance, further investigation could explore how selfcare practices relate to culture, especially in settings where traditional medicines are central to self-care for NCDs. In addition, future research should explore providers’ perspectives on the use of traditional medicines for self-care.

This study also has strengths. Our sample includes a balance of men and women with hypertension, diabetes, or both conditions across three different types of health facilities, enhancing its external validity. Second, our analytic strategy involved a multi-disciplinary coding team, consensus meetings about code structure, and reviews by all investigators, which facilitated both emergence of divergent interpretations and cross-coder reliability. Finally, this work generates novel directions for interventions to promote self-care for NCDs in low-income, rural settings.

## Conclusion

Conventional prescribed medicines were a key resource for self-care among patients living with diabetes and hypertension in rural Uganda. However, patients faced uncertain and inconsistent access to these medicines, which affected their choice of self-care practices. Patients drew on networks of social support among families and peers to reduce uncertainty and engage in mutual aid. Interventions to improve self-care and patient outcomes in rural LMIC settings may be more effective if these interventions strengthen access to medicines and leverage existing support networks.

## Additional File

The additional file for this article can be found as follows:

10.5334/aogh.3308.s1 Appendix 1. In-depth interview guide.
