# Supplementary material for: Social Support for Self-Care: Patient Strategies for Managing Diabetes and Hypertension in Rural Uganda
Source: Ann Glob Health. 2021 Aug 19;87(1):86. doi: 10.5334/aogh.3308 (PMC8378074; doi:10.5334/aogh.3308)
Supplement: Appendix 1. — In-depth interview guide. [file agh-87-1-3308-s1.pdf]

**In-depth interview guide**

**Introductory statement:** We are gathered to discuss about our health. I would like you to share with me how you take care of yourself and what you do or avoid to do to keep in good health.

1. Could you please share with me how you got to know that you had hypertension or diabetes? (Probe: How did you know? What was happening in your life then/ symptoms?)
2. I would like to know your thoughts about living with diabetes or hypertension. How do you view this condition? (Follow-up question: do you think you have enough information about this disease? Probe: what is the source of information?)
3. Let's talk about the symptoms / signs that you face as patients with diabetes or hypertension: What are some of the symptoms? how do you recognize them; how do you respond / react to them when they occur? [*what meaning is placed on these practice or response*]. (What about complications? What do you know about the complications that can affect you?)
4. Now let's talk about the medical care you receive at this facility: how often are you supposed to attend this facility? (Are you able to come for al appointments? Probe why).
5. We will talk more about the medicines you receive at the facility: Do you often get explanations for the medicines you receive? (probe: frequency of obtaining prescribed medicines, what is done when medicines are not obtained at facility).
6. What are some of the challenges you face when attempting to adhere to medicines prescribed or, even those you have obtained? (Probe: How easy or difficult or how easy is it for the patient to follow the recommended uptake of medicines give/ dosage
7. Apart from the medicines, is there anything else you do to be well, live happily or prevent adverse conditions? (Probe: what is done and why). Probe alternatives for medical treatment like herbal medication, prayers, social /family support, societal influences.
8. We will now talk more about our diet: share with me your feeding practices ever since you were diagnosed with diabetes (Probe: what foods and drinks do you often eat/take? (Probe: Why the food/ drink, alcohol consumption, smoking and salt intake awareness and practice).
9. Let's talk about exercises. Do you think these are necessary, please explain? (probe: Practices with regard to regular physical exercise importance of exercises, what they do or avoid to do to achieve physical activity?)
10. What are some of the other things (apart from diet and exercise) that the nurses or doctors have told you to do or to avoid? (probe: Have you been able to perform them? why not able to perform some recommendation? and why not able to perform other recommendation?) Probe about monitoring of the disease, foot care,

11. What other issues or challenges do you experience when keeping up to maintain good health? (Probe: social /family support, societal influences, receipt of care from community health workers)
12. What do you think can be done to best improve your uptake of healthy behaviors recommended by the health workers?

Thank you
